# American Society of Dental Surgeons, and American Dental Convention

**Published:** 1855-10

**Authors:** 


### Proceedings of Societies.

### AMERICAN SOCIETY OF DENTAL SURGEONS, AND AMERICAN DENTAL CONVENTION.

We publish the proceedings of the American Society as furnished by the Secretary, Dr. C. Bonsall, and am indebted to the kindness of Messrs. Jones, White, and McCurdy for a proof sheet of the report of the proceedings of the American Dental Convention, which, being more full in detail of practice than the report of the proceedings recorded by the Secretary, we give it to our readers.

The American Society met in Philadelphia, August 1st, 1855, at 10, A. M., in pursuance of a call from the President, which he had been requested to issue by the members present at the meeting in Cincinnati in May last.

The following members were present, viz: Drs. E. Townsend, J. S. Clark, D. R. Parmley, Cone, Dunning, Miller, Allen, Arthur, Goddard, and Bonsall.

The President called the attention of the members to the special business for which the meeting had been called, being "to take into consideration the propriety of dissolving the Society," which was discussed by Drs. Arthur, Dunning and Townsend; after which, for the purpose of bringing the subject before the Society, it was moved by Dr. Goddard, and seconded by Dr. Dunning, "That the American Society be now dissolved." The subject was then further discussed by Drs. Goddard, Clark, Miller, Cone, Parmley, Arthur, Townsend, and Dunning, when, on motion, it was resolved, that the subject under discussion be referred to a committee of five, consisting of Drs. Townsend, Arthur, Dunning, Clark, and Cone, who shall report upon the expediency of dissolving this Society at 5 o'clock, this afternoon.

Adjourned to meet at 4½ P. M.

AFTERNOON SESSION,

Met according to adjournment, when the following report was received from the committee:

*To the American Society of Dental Surgeons:*

Your Committee to whom was referred the subject of the propriety of dissolving this Association, after mature deliberation, have thought it inexpedient to dissolve the Society at present, and ask of the Society leave to report at the Annual meeting of 1856.

E. Townsend,

E. J. Dunning,

C. O. Cone,

R. Arthur,

John S. Clark.

The report was approved, and, on motion of Dr. Goddard, it was

*Resolved*, That said committee be further instructed, in case they consider it necessary for the further advancement of this Association, so to amend the Constitution and By-Laws of the Society as to alter the sum to be paid for initiation and annual dues as will best comport to the welfare of this Society, and do all other things in the premises, so as to perpetuate and place this Association upon a permanent basis, and to suit the views of the Profession generally.

This resolution was carried unanimously, and in consideration of there having been already two meetings held this year, it was thought best to postpone the regular annual meeting for the present year, and the Society adjourned to meet at the Astor House in the city of New York, at 10, A. M. of the first Tuesday in August, 1856.

Charles Bonsall,
*Secretary pro tem.*

*Report of the organization and proceedings of the first meeting of the "American Dental Convention," held in Philadelphia on the second, third and fourth of August,* 1855.

In pursuance of a call signed by some sixty of the dentists of Philadelphia and adjacent places, and published, by request, in the *Dental News Letter* for July, 1855, a meeting of a large number of the profession was held as above.

As it may be of interest to many, we give the names, so far as we were able to obtain them, of those present at the sittings of the convention, some eighty of whom signed the articles of the Association.

Drs. R. Arther, Elisha Townsend, J. D. White, D. Neall, J. Gillams, H. S. Burr, J. F. B. Flagg,'S. Dillingham, J. H. McQuillen, Edward Townsend, Jos. E. Parker, T. L. Buckingham, C. B. Foster, D. B. Wipple, S. L. Mintzer, E. M. Neall, J.S. Gillams, J.M. Harris, A.R. Johnson, D. Roberts, J. H. Githens, S. Roberts, C. A. Du Buchet, C. N. Pierce, J. S. O'Neil, S. Walton. A. M. Asay, S. S. White, W. Calvert, J. M. Barston, W. R. White, R. A. Porter, W. H. Clark, J. R. McCurdy, J. J. Griffith, L. Llamozas, A. L. Heston, T. G. Armstrong, J. Hayhurst, of Philabelphia; Drs. E. Parry, J. McCalla, S. Welchens, J. Walan, of Lancaster, Pa.; Dr. Jas. Fleming, of Harrisburg, Pa.; Drs. Jesse C. Green, E. P. Worral, C. M. Valentine, Westchester, Pa.; Dr. Jas. Lock, Williamsport, Pa.; Dr. H. M. Martin, Jersey Shore, Pa.; Dr. Chas. Moore, Pottstown, Pa.; Dr. D. S. Hutchinson, Hollidaysburg, Pa.; Drs. J. B. Rich D. R. Parmly, E. J. Duning, G. E. Hews, John Allen, C. W. Ballard, W. Crowell, New-York city; Dr. Fenn, Rochester, N. Y.; Dr. W. H.Dwnielle, Cazenovia, N. Y.; E. D. Fuller, Peekskill, W. A. Palmer, Poughkeepsie, N. Y.; Drs. S. P. Miller, and H. S. Bishop, Worcester, Mass.; Dr. F. Searl, Springfield, Mass.; Drs. Bonsall, Cameron and Hunter, Cincinnati, Ohio; Dr. Young, Zanesville, Ohio; Dr. C. O. Cone, Baltimore, Md.; Dr. W.H. Godeard, Louisville, Ky.; Dr. John S. Clark, New Orleans, La.; Dr. Kennedy, Wilmington, N. C.; Dr. P. Babcock, Raleigh, N. C.; Drs. Munson and McFarlan, Washington, D. C.; Dr. V. Shinn, Georgetown, D. C.; Drs. Kingsbury and Brown, of Mt. Holly, Marshall, of Camden, Harbert, of Salem, and Whittaker, of Bridgeton, N.J.; Dr. W. Potter, Norwich, Connecticut.

The meeting came to order at 10 o'clock, A. M., by the appointment of a temporary Chairman and Secretary, when, on motion of Dr. Elisha Townsend, a committee was appointed to nominate officers for a permanent organization.

*Committee.*—Drs. J. McCalla, of Pa.; G. E. Haws, N. Y.; S. P. Miller, Mass., Potter, Conn.; Bonsall, Ohio, Munson, D. C.; Brown, N. J.; Goddard, Ky.; Garrett, Del.; C. O. Cone, Md.; and, on motion of the chairman, Dr. J. S. Clark, of La., was added to the committee.

The committee reported the names of Dr. J. B. Rich, of New-York city, for President, and Dr. Chas. Bonsall, of Cincinnati, for Recording Secretary, which report was unanimously adopted, and the gentlemen took their seats.

On motion of Dr. J. S. Clark, a committe of one from each state was appointed to report a plan, with articles of association, for the action of the convention, and the following gentlemen were appointed as that committee:—Drs. J.S. Clarke, of La.; Munson, D. C.; Garrett, Del.; Goddard, Ky.; Cameron, Ohio; Marshall, N. J.; Potter, Conn.; Miller, Mass.; Haws, N. Y., and Elisha Townsend, Pa.

After a short delay the committee reported the following preamble and "articles of association," which, on motion of Dr. Dwinelle, were taken up for consideration by articles, and after being very slightly amended were adopted, as follows:*

"At a national convention of dentist, assembled in Philadelphia on the second day of August, 1855, for the purpose of consultation upon the measures best adapted to advance the science, and common interest and honor of the profession, the following plan of organization was adopted:

Art. I. The association shall be called the American Dental Convention.

Art. II. The association is intended to promote professional and personal intercourse among those who are engaged in the cultivation and practice of dentistry throughout the world; to advance the cause of dental education, and systematize and strengthen the exertions of its friends, and, by a mutual interchange of opinions and experience, to advance the knowledge and liberalize the relations of the members.

Art. III. The convention shall consist of the members of the convention who shall sign these articles of association, and of such other practitioners of denistry and auxiliary branches of science as shall hereafter be elected to membership, and in like manner sign these articles.

*The title in the report was American Dental "Congress," which subsequently, on motion of Dr. Arthur, and after some discussion, was changed to Convention.

Art. IV. Candidates for membership shall be nominated by a member of this convention, at any of its meetings, and every such candidate as shall receive a majority of the votes cast upon the question of his admission, shall be declared duly elected.

Art. V. The stated meetings of this convention shall be held once every year, on such day and at such place as the convention shall at, each session, appoint for the next meeting.

Art. VI. The officers of this convetion shall be a President, Vice President, Recording Secretary and Corresponding Secretary, all of whom shall be elected to serve for the ensuing year, on the first day of the annual session, or on such other day of the session as may be appointed by the convention. And the officers incumbent shall hold their offices and exercise the functions thereof until their several successors shall be elected and installed.

Art. VII. The Vice President shall in the absence of the President, or in the event of his death or resignation, perform all the duties of the President, and in the absenec, or death or resignation of both President and Vice President, the Corresponding Secretary shall serve as President, *pro tempore.*

Art. VIII. All elections shall be determined by majority of the votes cast by the members present, and the manner of voting shall be either *viva voce* or by ballot, as the convention shall at the time determine.

Art. IX. A committee to prepare business for the session shall be appointed by the President elected for the year then expiring, which committee shall consist of one member from each state represented.

Art. X. The Business Committee, provided for in Art. IX., shall be the standing committee of reference for all essays and papers proposed to be read by members before the convention, and shall report their number, subject and length to the President, with their advise as to the order most expedient to be observed in presenting them to the convention, and the President shall thereupon appoint, at his discretion, the time at which they shall be read: *Provided,* that it shall always be competent to the convention to assign, by resolution, any other time for the reading of such papers, or to postpone such reading indefinitely. And the convention may also, by resolution, order the reading of any paper or communication at such time as it shall deem expedient.

Art. XI. All papers read before the convention by the members shall be the property, and at the disposal of their authors, unless otherwise disposed of by resolution of the convention with the consent of the authors.

Art. XII. The President may appoint any member or members of the convention to read a paper or papers upon any professional subject, at any subsequent session.

Art. XIII. The convention shall order and determine all matters not herein provided for, by resolution.

Art. XIV. The funds of the convention shall be held and appropriated by the Recording Secretary to the discharge of its expenses, and he shall report his accounts to the convention on the last day of the session, and shall assess a *pro rato* tax upon the members present to defray the same.

Art. XV. The President shall, on the first day of the session held at the close of his term of office, organize the session, direct the reading of the minutes of the last session, and deliver an address before the convention upon such subjects as he may deem most useful and important for their consideration.

Art. XVI. These articles may be altered and amended in whole or in part at any session of the convention, by a majority of the votes of the members present."

After the adoption of the articles of association, on motion of Dr. E. Parry, the convention resolved itself into the "American Dental Convention," and the former officers were re-elected vive voce, and Dr. J. H. McQuillen, of Philadelphia, elected Corresponding Secretary, by ballot, and Dr. J. S. Clark, of Louisana, Vice President.

Adjourned to 4 o'clock, P. M.

AFTERNOON SESSION.

Convention met at 4 o'clock, according to adjounment.

The Chair stated that it had been proposed to occupy the present session in professional discussion.

Dr. Townsend made some remarks on the subject of the preparation of gold for filling teeth, and gave some reminiscences and modes of practice, such as rolling gold into pellets of different sizes; uses pellets and folds, and strips of numbers 10 and 30, the thinner the walls and larger the cavity the thinner the number of the gold. Had been told of the form of cylinders, but rather adhered to the use of pellets and folds; alluded to Dr. J. S. Clark as using the cylinders.

Dr. J. S. Clark (of New Orleans) described his method of introducing gold in the shape of cylinders, and manifested great earnestness and fervency on the subject. Said it was one of the few things that, although its claims rested on purely scientific principles, and offered a beautiful theory, it was so much more interesting and beautiful in its practical illustration that, one practical test, was worth all the description that could be given.

The old and familiar experiment of producing cohesion, by placing two plane surfaces in contact and forcing out the air as in the experiment with two leaden bullets, (with planes cut on each and forced together,) will explain the principle of cylinder filling. It is nothing more or less than taking advantage of this principle in forming a filling from gold foil.

The foil is carefully rolled into cylinders, as a bolt of cloth is rolled, and of a length to suit the depth of the cavity, and intended it to be a little longer than the cavity is deep. They are rolled of all sizes, from a full sheet down to the size of cambric needle wire. Some are made conical in shape, but most are plain, straight cylinders. Some are rolled very lightly, others quite hard. They are formed by folding the sheet, or part of a sheet of foil, into strips, as *wide* as the cylinder is to be *long*. This strip is then rolled on the point of a very fine (five-side) instrument, and the strip cut off when the desired size is attained.

The application is to place soft (or lightly rolled) ones in the cavity endwise, being careful that each cylinder reaches the bottom of the cavity and protudes a little outside the orifice. When the cavity is apparently full, a round instrument is passed down between them, and another cylinder (a little harder rolled) is forced up. This is repeated, decreasing in size of instrument and cylinder, (the latter of increased hardness,) until no more can be introduced. At this point the filling will be found to be one combined mass of gold, which can be cut and filed into shape, and polished as perfectly as any piece of molten gold, for the cylinders being, when introduced, all ventilated, or open at the end, and of plain surface in contact, by this manipulation the air has been forced out, and cohesion taken place according to the natural laws of cohesion.

It is well known that all cavities are not of regular shaft-shape. All variations as to shape are easily filled in this way, by using cone-shaped cylinders, with the base of the cone in or out, as the shape may demand. A cavity with but two opposing walls, and they standing at nearly right-angles, can be filled by springing an arch from one wall to the other. Besides many other advantages, the operator will always be sure of finding the orifice or margin of gold perfect, as to fullness and adaptation.

I have used gold in this way almost exclusively for five years. Showing it at the time to Dr. Friedrichs and Dr. Jas. S. Knapp, of New Orleans, who have adopted it fully, and of whose operations since, the profession have been pleased to speak in the highest terms, but not more than such operations always deserve. Subsequently, I have shown it to several, who have adopted it; and among those who have tested it for three and four years are Dr. C. W. Spalding and Dr. H. J. B. McKellops, of St. Louis. Names are mentioned, that their operations may be noted as they come under the eye of the profession.

Dr. Townsend did not wish to occupy time, but would state that the greatest difficulty with him, was the preparation of a perfect cavity; and he was compelled to say, after putting in a filling, "that would be a perfect filling if." Alluded to the difficulty of getting every thing into proper condition, and with proper finish.

Professor Author had listened with great interest to the remarks on this subject. The method described by Dr. Clark, he thought eminently fitted to accomplish a great object in filling teeth, the union between the material used for filling, and the periphery of the orifice of the cavity. He had always regarded this as one of the most important if not the most important feature of the whole operation. The proper preparation of gold for the purpose in question, had always been a subject of great interest to him, and he had for years past experimented a great deal in various ways, to reach the best method. The defect of gold foil, with all its great advantages, had always been a source of trouble to him, and he had been anxious to find some way of overcoming these defects. There was no question, in his estimation, that a great deal would be gained if the different portions of the gold placed in the cavity could be readily made to adhere together, and he had steadily looked forward toward this desideratum. Some years ago he had propsed to use heavy numbers of foil, cut into single strips, and condensed with a fine-pointed instrument. In this way, by bringing great pressure upon a very small surface, the different parts were made to adhere; or, if no adhesion actually take place, one layer was forced into the other at so many points, that it simulated adhesion. This Prof. A. at the time regarded as an advance upon the ordinary method of using foil, and only abandoned it after having used it for more than a year, on account of the difficulty of getting suitable instruments made, as the necessary instruments being fine and hard were easily broken, and their frequent renewal involved so great a loss of time, that the advantage gained was not a conpemsation for it.

When crystalized gold was introduced, Prof. A. had taken hold of it with great avidity. It gave greater promise than any thing he had previously met with. He had used it almost exclusively for more than a year, and after becoming accustomed to its use, found it of great service. He had undoubtedly found difficulties attending its use.

The President requested Prof. A. to state to the convention the nature of the difficulties he had encountered, in making use of crystalized gold.

Prof. A. was quite willing to state explicitly the nature of the difficulties referred to, but he did not wish to trespass. He would say, however, that he had found it necessary that the material should be kept perfectly dry—mosture, he fouud was fatal to its adhesion; and many cases occur where it is exceedingly difficult to exclude mosture entirely. A great difficulty which he had encountered was from the want of uniformity in the material itself. From the same manufactures, so far as his experience in its use had gone, it was impossible to get two specimens precisely alike. At one time the article was all that could be desired, and the next lot, perhaps, would be greatly inferior. He had found, also, that it required a greater amount of time to make a reliable filling in many cases, with this material, than with gold foil, as commonly used. It could not be placed in a cavity in large quantities and condensed with large instruments, but it was necessary to use it in small portions and to condense it with very small instruments. But in the face of these difficulties, Prof. A. had no hesitation in declaring that the results, in his own practice, fully compensated him for the labor he gave to it; and he had been quite willing to devote to it the necessary labor for the results he was able to obtain.

But, in making some experiments to ascertain the relative density between a filling made of gold foil and of crystalized gold, he was led to the discovery that gold foil could be used in precisely the same manner as crystalized gold. [Here Prof. A. described the method of using gold foil, an account of which has already appeared in the "News Letter."]

Dr. Neall remarked, that the cohesion of gold was no new doctrine, but he had not thoroughly comprehended Dr. Clark's method of filling a cavity with cylinders. He (Dr. N.) proposed to make each part of his filling cohere as he introduced it, and not depend upon one portion of the gold packing the other, as he implied must be the case, if cylinders were set up en masse in a cavity, and then compressed.

Interrupted by the President, who stated that remarks must not become discussional on personal methods, but that each, in speaking, should confine himself wholly to the explanation of his own method.

Dr. J. M. Harris gave his experience, and various methods of preparing gold for fillings.

Dr. Neall asked for explanation, how Dr. Clark would proceed in filling very angular cavities with cylinders.

Dr. Rich remarked, that his preceptor in this country, Dr. Park, taught him to prepare his gold in cylinders, and formed them by rolling the gold over a watch spring; he then went on to give the method of introducing the gold; described a plan of forming an open coil of gold, the interstices of which were filled with cylinders. He objected to the wedge-shaped instruments, and thought them entirely unsuitable; he used straight, thin points, flat but not wedge-shape. Alluded to crystal gold fillings of recent date as promising decided improvement in that direction, having tested it thoroughly, by working it in water, saliva, flour and other similar substances, yet without impairing its adhesiveness, or preventing its being formed into a solid plug.

Dr. Dwinelle remarked, that he considered gold foil one of the most valuable agents the profession ever had any thing to do with. He did not wish to say any thing *against* foil, but to speak in favor of crystal gold. He alluded to his connexion with the introduction and improvement of crystal gold; he thought the ultimatum had been reached, and was fortified in his assertion by the severe tests it had been subjected to by himself and others. It had stood the test. He had failed in some cases of course, all of which, however, were to be attributed to imperfect manipulation. He considered that an established fact could never be gainsaid, however humble its author might be; a successful operation proves not only its own success, but that it is possible for every one to be equally successful. He believed that many important facts have been established in favor of crystal gold, and that unparalelled success had been gained by its use. He spoke of its superiority over foil in many cases, in that it was capable of being built up into independent forms, and its particles thoroughly integrated together into an absolutely solid mass, which would not absorb moisture, and whose specific gravity was equal to solid gold, as had been repeatedly proven by actual experiment. He referred to stoppings of crystal gold which had been worn in the mouth for two years without change; alluded to decided improvements, which he said had been recently made by the manufacturer; and that an entire uniformity in quality could always be relied upon in the future. Its successful use required time, care and experience. Used soft paper in drying out the cavity. Would prefer annealing gold foil without permitting the flame to come in contact with the gold.

Dr. Kingsbury was gratified at having the pleasure of meeting the profession, as he was desirous of learning and imparting, if in his power. Had tried sponge gold in some cases; thought he had made very good fillings; but in the great majority of cases had been able to make better fillings with gold foil, No. 4. Attributed the harshness of foil to friction between the leaves of the book. Had tried annealing on using the foil, and thought well of it. His confidence was greater in good gold foil than in sponge gold.

Dr. S. P. Miller commenced practice with the use of ribbons, folded with a thin spatula; had tried gold in all the various forms offered to the profession; did not condemn the form of cylinders, they being sometimes used, but did not find them equally applicable to differently shaped cavities as pellets. His want of universal success, claimed by others, may have been owing to the fact that he had not so perseveringly continued their use. Much depends on habit; has had the greatest success with pellets of different forms—round, oblong and flattened, with serrated instruments for packing, commencing to fill with pellets, and finishing with ribbons or small ropes. The *oblong* pellets take the place of cylinders, being more easily kept in place. The ribbons of various thicknesses and widths, cut in square blocks, is another excellent form for protecting the thin shell of a front tooth; is not confined to any one form for all cases, but uses flattened pellets—the round pellet slightly pinched between the thumb and finger—more than any other. His experience with sponge gold had been unfavorable; he had found it uneven in quality, and not to retain its color, owing to the retention of impurities in the process of its manufacture; but if the preparation has been so greatly improved as we have just been told, then some objections to its use have been removed. While he did not doubt what he had just heard about sponge fillings, yet, he must say, he had not been so fortunate as to produce the same wonderful results, and have the same success claimed for it, as those who had advocated it so strongly.

Dr. J. D. White, humorously remarked that he supposed the cavity now all prepared and dry, ready for filling, and as sponge gold had been tested in so many ways, and with so many other substances, and with so many results, he would suggest the addition of some "cod liver oil" as calculated to make it much better and sustain its reputation.*

Dr. J. F. B. Flagg, had tried sponge gold, and his experience was adverse to it. The manufacturers, to judge from their advertisements, seem to expect from the dentist a term of probation in the use of a poor article, before they could trust them with the very superior article, of which we have just been informed. But the article had disappointed the profession, and he must term the course pursued by the manufacturers' humbug, if this new material is what was promised.† [Called to order by the chair.]

Dr. E. Parry gave his experience with foil, which had served his purpose satisfactorily; alluded to its occasional harshness, which he believed was caused by friction in transmission, and that it deteriorated by absorbing moisture, and was of the opinion that by annealing, its texture and ductility were restored.

On motion, the convention adjourned to meet the next morning at 9½ o'clock, A. M.

---

* His views on sponge gold have already been given at large in the page of the *News Letter*.

†PHILADELPHIA, Sept. 13, 1855.

DR. MCCURDY—*Deay Sir*:—In an interview with you recently, I understood you that it was the desire of our friend Dr. Dwinelle, that I should withhold, or modify in some way, the expression made use of by me, in reference to *sponge gold*, at the late convention held in this city. As it affords me an opportunity of explanation, I will embrace it. If a true re-

FRIDAY MORNING, August 3.

Association met at 9½ o'clock, President in the chair.

After the meeting had come to order, Dr. Arthur made a motion to amend the first article of the association, by inserting the word "Convention" instead of "Congress," and he was, on motion, allowed twenty minutes to give his reasons, which he proceeded to do. The debate was participated in by Drs. Townsend, Flagg, McQuillen, Kingsbury, Dwinelle, Munson, after which the amendment carried, almost unanimously.

Dr. Townsend then suggested, as a fit subject for one hour's discussion, the best method of keeping the mouth and cavity in the tooth perfectly dry during the operation of filling, and gave his method, which was by the use of paper.

Dr. Rich's method of keeping the cavity dry was by the use of paper. He remarked that paper slightly sized will absorb very quickly, and ordinary tissue paper rubbed until the sizing is broken up, and then rolled into rope was a useful application. In the use of napkins, he folded and applied to the lower jaw to absorb the saliva from the ducts, and used them also in other positions; for the ducts of upper jaw used ordinary blotting paper, which he made to adhere to the mouths of the ducts; by such and similar appliances had been enabled to keep the mouth dry for an hour. The paper leaves me nothing to desire. I never have a case in which the water encroaches on me in three-quarters of an hour, I care not how wet the mouth may be.

The President stated that he would now adopt the rule of calling upon the members individually for their experience upon this subject.

Dr. J. M. Harris had used paper, had filled the opposite duct with a pledget of cotton, and used cotton dipped in fine plaster of Paris.

Dr. Ballard had used paper, as described by the President,

---

port is made of those proceedings, it will be seen that *two* gentlemen had preceded myself, and spoken "their fill" in praise of this new agent. In following, I was so unfortunate as to *prefix my fears* to the relation of *my* experience, and the moment I feared a "*humbug*" I was cut short by a head; fairly decapitated, and my speech, half made up, suffered to hang upon this coarse but very expressive word.

It seems to me that the friends of sponge gold must, at present, be held responsible for *any* meaning that may attach to that expression, and, as I have now consumed the space you say you can afford me in your present number, I suppose I must wait another three months, for a suitable opportunity to give my views. Yours, &c., J. F. B. FLAGG.

but of a thicker character. It is known as French bibulous paper, and is manufactured in France and Switzerland for the purpose of protecting from moisture the watch movements, which are exported from those countries in immense numbers.

Dr. Dwinelle would endorse what the President had stated on this subject, and had followed the same mode of practice. Spoke of the absorption of the broken fibre of the paper by capillary attraction. Alluded to the leakage of serum throuh the tubuli, and referred to discoloration occurring behind the best of fillings, sometimes; queried whether it was occasioned by exudation of vitiated fluids from dentinal tubuli.

Dr. Clark was indebted to the journals for the suggestion of paper. Had used subsequently lithographic paper softened by rubbing; had found it very useful.

Dr. McQuillen, in the first years of his practice, used finely carded cotton, but, at the suggestion of Dr. Townsend, adopted tissue paper; has tried both thick and thin; for general purposes prefers the former, but in absorbing the moisture from the pulp cavity, employs the thin, which, when torn into narrow strips, parallel with the fibres, and rolled tightly, acquires a roughness and flexibility that admits of its being passed a considerable distance up the root; his attention was directed to this fact by Dr. D. Neall. To protect the tooth operated on, from the salivary and mucous secretions, placed strips of muslin (freed from sizing) between the cheek and gum; in addition, when operating on the lower teeth, enveloped the tongue with a small and soft napkin.

Dr. Flagg.—In regard to tissue paper and cotton, we must consider the principle upon which it acts. The sizing he thought very necessary in absorption. Had used strips of fine cotton cambric, which, after washing and starching, he found would draw off the moisture in a very satisfactory manner.

Dr. S. P. Miller, had used prepared flax, paper, also cotton, deprived of its oil by boiling in a solution of potash, for drying cavities.

Dr. Kingsbury had used tissue paper for about eight years, which was communicated to him by Dr. Townsend. Used napkins upon the inside and between the gum and cheek. Had found some difficulty in keeping the cavity dry when situated near the gum; had passed strips between the teeth, and used gum-elastic to keep off the edge of the gum from the tooth. Had thought paper, moistened and then dried, would absorb more rapidly.

Dr. Buckingham remarked that, in keeping the margin of gum away from the teeth, he had used soft wood; used paper and also cotton.

Dr. J. D. White stated that, in applying napkins, he had formed them by folding them around a watch spring, or a strip of sheet lead from two to three inches wide, so that when applied between the teeth or gum and cheek, they would retain the shape desired. Had invariably succeeded in keeping the mouth dry with a fixture of this kind; regretted he had not brought it with him to illustrate his method.

He had used bibulous paper in drying out the cavity, and would return his thanks to Dr. Ballard for the specimen sent him.

Dr. W. H. Clark had always used lint, and with satisfaction.

Dr. S. L. Mintzer used napkins and cotton, and strips of muslin; was in favor of the use of plaster of paris. Had found the saliva pump, invented by Dr. Arthur, a very useful contrivance.

Dr. Fuller, in cavities in close approximation to the gum, had used cotion twine passed between the teeth and forced down between the edge of the gum and the tooth; had used napkins, much after the manner already described, rolled over watch spring, whale-bone, etc.

Dr. H. H. Martin used napkins, cotton, etc., and scraped the cavity just before the application of gold.

Dr. Kingsbury had used sponge about "the size of a piece of chalk."

Dr. Clark wished to say, in evidence of the benefit of the convention, that he had received one idea, which although a small thing, he felt was worth a pilgrimage of two thousand miles. He was ignorant of the originator of it, but would like to see him and thank him for it.

It was the simple use of a dove-tailed wedge of soft, close-grained wood, passed between the teeth, pressing down the point of gum, (when filling approximal cavities,) arresting hemorrhage and enabling us to operate by sight in that part of the cavity, instead of on suspicion, as well as saving the patient much pain in filing, polishing, &c.

Dr. Townsend had been taught the use of napkins by Dr.

Hudson, who was very particular in this matter, and who used them of a suitable size and character. The use of cotton he had abandoned, for the purpose of drying the cavity, but used it in wiping out the debris in the cavity after excavating. Used wedges of wood to press the gum away, but not to penetrate the gum.

Dr. S. P. Miller alluded to the secretions of the mouth in the treatment of approximal cavities, and the great necessity for keeping these surfaces free and clean. In excavating cavities below the margin of the gum, had used chloroform, morphia and tanin, applied on cotton, as astringents.

Dr. J. D. White, by request, described a little apparatus he used on his finger in keeping the tongue away, in filling lower cavities. He gave the idea by forming a piece of paper somewhat of the character of a ring, to go around the finger, from which a shaft extended parallel with the finger some one and a half inches, on the end of which a circular piece was attached about the size of a ten cent piece, at right angles with the shaft.

To an inquiry put by Dr. Clark, he explained the use he made of tin foil in forming wedges to press the gum away; had used cotton for the same purpose, forcing it down between the gum and tooth.

Dr. Townsend alluded to the difficulty in keeping the cavities dry on the anterior surfaces of the front teeth. In some cases had used caustics to produce a slough, then, by removing the gum, was enabled to get at the cavity, but always preferred a wedge where applicable.

Dr. J. M. Harris hoped the profession would make trial of the plaster of paris, with a view of preventing the discoloration of teeth after having been filled. He was disposed to think favorably of it.

Dr. J. Fleming had used plaster on cotton, after the cavity was prepared, with a view of closing up the tubuli; wiped the cavity well with the cotton and plaster on the point of an excavator.

Dr. Clark had removed a filling that had been in nearly a score of years, and found, in the bottom of the cavity, dry plaster of paris.

Dr. S. P. Miller hoped too mnch would not be left in the cavities at the expense of sponge gold or gold foil.

Dr. Kingsbury could not understand how plaster would answer the purpose, as the current through the tubuli is from the outside toward the centre.

Dr. Townsend wished to explain that, in putting in a wedge, "he cut off both ends of the wood."

On motion a half hour was devoted to the hearing of cases.

Dr. Dwinelle had had a case which interested him, and which exhibited the ignorance of medical men on the subject of dentistry. Dr. Munson, of Washington, had extracted the teeth of a lady of Washington, and for whom he had inserted a full set of teeth. After wearing the plate a short time, she was annoyed by the appearance of a tumor on the roof of the mouth, for which she had been under medical treatment, and without benefit. The tumor was thought to be of a scirrhous character by her medical attendance. On examination, discovered an opening in the palatine arch, far back, about the size of the head of a pin, the inflammation involving the whole palatine arch. He made broad incisions crosswise, in the hope of finding the cause of all the trouble, when he discovered the point of a bright object, which, on further dissection, proved to be a tooth, and on applying the forceps, extracted an immense cuspidati tooth, which had been lying in an oblique direction, imbedded in the palatine arch and extending into the nasal process, the tooth perfect in form, but diseased with exostosis at the end of the fang.* Gave the particulars of a similar case which had been related to him by a friend.

Dr. Clark alluded to the case of a lady from Belleville, Ill., (age 44,) for whom he inserted a plate in 1840, and finding the plate subsequently thrown up from the gum, and a bright spot on the under side of that part that covered the apex of the palatine arch, found on examination a small cuspidati tooth protruding.

Dr. Searle exhibited a right upper lateral incisor, which he found in nearly an inverted position—the apex of the root lying between the roots of the front incisor and cuspidatus—the biting edge extending upward and backward above the junction of the maxillary and palate bones, the anterior plate of the enamel being towards the palate, and much wasted by absorption; the posterior nearly perfect. Alveolar abscess had existed for many years. A patient, 28 years of age, had suffered much from St. Vitus' Dance and spasms. Three months since the tooth was removed, and, as yet, no return of St. Vitus' Dance or spasms.

*The position of the tooth was made very clear by diagrams upon the black-board.

He had also removed a right inferior dens sapientia which was *entirely inverted*, and had been the cause of much suffering and anxiety for eighteen years. The tooth was deeply imbedded, the maxilla being much enlarged. Alveolar abcess had existed for twelve or fifteen years, discharging into the mouth. Physicians had called it a case of caries of the maxilla. The discharge ceased in a few weeks after the removal of the tooth, but the enlargement seems to be permanent.

On a call being made, the proceedings of the previous day were read, with the articles of the association, and the minutes approved. When, on motion, adjourned to meet at 3½ o'clock, P. M.

At 3½ o'clock, the President called the meeting to order, after which it was proposed to occupy an half an hour in discussing the subject of "allaying the sensibility of dentine in preparing cavities in the teeth for filling."

Dr. Clark used nothing to destroy the sensibility of dentine. He dared not do it, having seen so many cases where the vitality of the tooth had been destroyed through an eighth of an inch of solid dentine. Mentioned a case of a lady for whom he operated this season, who had seven destroyed in that way, and all ulcerated, when the largest cavity when filled was no larger than No. 6 shot. He sometimes used astringents, but never escharotics.

Dr. Rich had used most of the substances suggested for the purpose, but had met with the greaest and safest success with ether, used in a certain way. Inhaling small quanties of ether whichentirely removed the sensibility, if administered carefully, was perfectly successful.

Dr. J. M. Harris had used arsenic occasionlly, also cobalt and other articles, but ether latterly and generally; spoke of its happy effects, giving illustrations.

Dr. McQuillen had foud arsenic efficacious in relieving excessive sensibility at the neck of the tooth. Pursued the plan proposed by professor White, of rubbing a short piece of dampened thread in dry arsenic, then tying it around the neck of the tooth for four or five hours when the application is removed, and the surface of bone, which it covered, is well pollished with pumice. Never employed arsenic to obtude the sensibility of dentine when preparing a cavity for filling, but used chloride of zinc in the state called butter of zinc, (prior to deliquescence,) when it is soft enough to be cut with the ordinary hatchet-shaped instrument, on the blade of which as small a portion is carried to the cavity as may be desired. To a question, replied that its application is nearly always attended with pain.

Dr. Flagg was in favor of using the arsenic, morphine and creosote in equal parts, but always prepared just before using and had found it very satisfactory. He had tried ether in some cases.

Dr. Searle had used chloride of zinc in but one instance, and on account of the pain produced, had abandoned it. He prefered *sympathy* and *encouragement* to *poison* or *caustics.*

Dr. Dwinelle—The subject interested him greatly, but his success had not been satisfactory; had abandoned the application of arsenic and cobalt. He had found that where it had been used, the teeth generally were injured or destroyed. He used temporary remedies, such as a strong solution of camphor, in repeated applications; also, chloroform, creosote, tannin and tannate of lead. He related an instance in which he used nitrare of silver, which latter he allowed to remain over night, and, on removing in the morning, he was able to operate with comfort. He explained his method of preparing and applying the arsenic.

Dr. Ballard was pleased to have the opportunity of laying before the meeting a new method of allying sensibillity. For the past eight months, and latterly with almost invariable success, he had used a combination of chloride of zinc and chloroform. He took a piece of the chloride of zinc about the size of a pea and covered it with cloroform, and used from the soft surface of the zinc. Applied the zinc while excavating, allowed it to drop against the exposed or recent surface of sensitive bone as the decayed matter was being removed. The sensation produced was that of heat. Had never known injury to result from its application. The greatest success with this preparation was met with when teeth of a soft character are treated; such teeth are usually the most sensitive. Teeth of a dense character require a longer time in the application before the effect is produced; seldom, however, exceeding two or three minutes. It was in only *very* dense yellow teeth that the application had failed in his hands, though *invariable* success could hardly be expected from any one method of treating teeth of any class. Had never been in the habit of using arsenic, but had seen its effects when applied by others, and considered it objectionable.

Dr. Kingsbury had used arsenic, but with bad results; had also used chloride of zinc, chloroform, gun cotton, etc. He removed the decayed portion as best he could, and then polished the surface of the cavity with pumice.

Dr. S. P. Miller had used arsenic in some cases with great success, also used other substances, as morphine and tannin in equal parts, moistened with creosote or chloroform; also used chloride of zinc with occasional benefit.

Dr. J. J. Griffith had found the greatest sensitiveness at the end of the tooth, where the dentine terminated; had a favorable opinion of arsenic.

Prof. Buckingham used chloride of zinc in as crystaline a form as he could obtain it, as a powerful absorbent.

Dr. James Fleming sharpened his excavators as sharply as possible, and then drew the point over a razor strop, and by careful and dexterous manipulation could generally manage the case with satisfaction.

Dr. John Allen had similar experience to give as the last speaker.

Dr. Kingsbury gave some further explanation of his method.

Dr. C. B. Foster had pursued the course of pracice alluded to by Dr. Flagg,—creosote, morphia and arsenic—and on applying it immediately in contact with the exposed nerve, permitting it to remain some three hours. Want of success may be owing possibly to the application of too much or in allowing it to remain too long.

Dr. H. H. Martin had used arsenic but without that success he had anticipated. Used chloride of zinc in its crystal form with success.

Dr. C. Moore had used arsenic, morphia and creosote for ten years, before using it had observed this longitudinal process of removing decayed bone, had applied the arsenic in the dry state from a sense of greater safety, and was of opinion it acted best in that form.

Dr. Neall used arsenic for destroying the nerve, and only when he intnded to extierpate that organ. Had for many years abandoned its use and that of all other common assuasives in obtuding sensibility of dentine. He employed (and with success almost absolutely invariable) this simple formula: To four inch fine Arkansas stone add six drops of sweet oil, rub in with the blade of a well-formed and well tempered excavator, applying the result, a fine keen edge, to the margin of the caries, and bearing well upon the healthy bone with steadiness and quantum suff. of patience, he could *whip out*, in a moment or two, the major and more sensitive parts of the decay, then proceed to form his cavity more deliberately. He always endeavored to cut as much as possible from center to circumference, and more toward than from him; putting his patient upon his legitimate endurance, and by a prompt and straight forward manner securing the like from him. He (Dr. N.) believed in the magnetism of backbone.

Dr. J. N. Parker could endorse what had been said by the last gentleman. In some cases had used arsenic with satisfactory results, but always preferred the principle of going to the root of the tree.

Dr. Mintzer only used arsenic when the nerve was exposed; used persuasion—remove a little and postpone further operation for a time.

Dr. Arther thought that a great deal might profitably be said upon this subject. He had made use of arsenic for the purpose, under discussion, and with good results—results so good as to lead him to say, without hesitation, that by observing very simple and easily understood precaution it might be used safely. In some case when he first began to use it, he was free to confess, bad results had followed its use, but he could, subsequently, easily account for these untoward results. His experience with it had since been of the most satisfactory character. He used it in the dry state and preferred it in the form of cobalt. Cobalt ore contained, he stated, as is well known, a large proportion of arsenic; in combination with cobalt, the arsenic appeared to be less soluble, and consequently less liable to pass through the dentine and effect the pulp injuriously. It was a mistake, he said, to suppose that after the lapse of months or years, arsenic applied in this way could finally reach the pulp unless it was applied in considerable quantity. The minute portion which might remain in the substance of the dentine after preparing a cavity, to which arsenic had been applied for the purpose in question, could not produce the result spoken of. Arsenious acid as it destroys the vitality of a tissue combines with it and becomes inert. For this reason, a given quantity is capable of destroying the vitality of only a given portion of the tissue of a tooth. If a cavity had reached the vicinity of the pulp, and enough arsenic were applied to reach and destroy its vitality, this would occur in a short time. If a few weeks elapsed without untoward symptoms he would not look for them subsequently. He regarded it as necessary that this agent should be used for the purpose of destroying the sensibility of the dentine with great caution but if due caution were observed, it might be employed, in certain cases, with great advantage both to patient and operator. Prof. A. described the method of employing this agent for the purpose under consideration, at some length.

Prof. E. Parry enumerated various articles which he had tried—nostrums ad serinum—until arsenic came up, which he had used with success.

Dr. Palmer had used arsenic for a number of years, in the smallest possible quantities, in combination with morphia, an objectional remedy but efficacious; viewed it in rather a favorable light.

Dr. O. Munson's experience agreed with many others in method of treatment.

Dr. W. H. Goddard had used all the remedies, yet not satisfied; thought Dr. Neall's method the best that had been suggested.

Prof. White thought five minutes too short to do justice to the subject; his confidence in arsenic was unshaken, and he spoke from an experience of seventeen years. It will destroy the sensibility; have used it in hundreds of cases; applied it dry spread over the whole surface, and could then cut away with the utmost satisfaction and without subsequent danger; used chloride of zinc where he found that substance which he would denominate corky; it gave a dull pain yet bearable, but after two or three applications the decay may be removed without any pain. Where arsenic had been allowed to remain too long it permeated the gum and then he had to treat the tooth for inflammation of the pulp; he used it alone in contact with the surface, it thus acts more promptly. It is impossible to use it without danger to the pulp cavity, therefore great care must be observed He here gave the risks; was careful to remove every particle; he never put it in a deep seated cavity.

Dr. James Locke, by request, explained by models his method of getting up casts or dies by a new form of flask; after which, on motion, the thanks of the convention were tendered him for the explanation given and for his liberality in bringing it before the profession.

On motion, an assessment of one dollar on those present was made to meet expenses of meeting.

On motion of Dr. Charles Moore, the place and time of meeting for the next year, was now considered, and decided in favor of the city of New-York, on the first Wednesday of August.

On motion, adjourned to meet next morning at 9 o'clock.

In the evening, the strangers in the city, by invitation of the Philadelphia members, partook of a collation at Pakinson's, at which many of the good things of life were discussed and appropriate sentiments given by Drs. Arthur, Dwinelle, Townsend, Rich, D. Neall, J. S. Clark, J. D. White, Ballard, Palmer and C. O. Cone

SATURDAY MORNING.

The convention came to order at 9½ o'clock. Minutes of the previous day's proceedings read and approved.

Dr. Townsend alluded to the danger of sometimes having a finger bruised in the mouth of the patient, and exhibited an appliance, in the form of a large gold signet ring, which he stated had been presented to him by the heirs of Dr. Flagg, of Boston; also exhibited two file carriers, of somewhat peculiar form, and carrying files of a V shape.

Dr. Gilliams would embrace the opportunity to apologize to Dr. Bonsall, for having thirty-five years ago hurt his feelings. Dr. Bonsall immediately explained the circumstance as follows: Thirty-five years ago he had called upon Dr. Gilliams for professional service, on which occasion the Dr. had extracted three teeth for him, one of which he had to split in removing, *which had hurt his feelings very much.*

Dr Flagg explained his method of taking plaster models either for correcting irregularities or getting his metallic casts. In the composition of casts, uses tin 3, lead 7, and bismuth 6 parts. Melt lead first then add the others.

Dr. Townsend, in a few remarks, alluded to the necessity of having to fight both the patient and family physician, because of pre-conceived notions and medical advice, and having to contend against these adverse influences, relating cases in illustration.

He considered filling teeth a very important matter, was rather adverse to wedging teeth apart; preferred filing, the spaces being they more easily kept clean. Alluded to having seen teeth filed by Dr. Gilliams forty years ago, and in good condition.

Dr. Gilliams gave his experience in filing teeth, remembered a case in which the patient in eating corn off the cob could only secure every other grain, this he thought a rather free use oft he file, but was favorable to the file, and used it freely.

Dr. S. P. Miller, in speaking on the same subject, related a case where the patient asked him if he "worked on the soft pressure system," stated that she had her teeth operated on a few weeks prior in New-York, on that system without hurting her, but that the fillings were all gone and she wished her teeth attended to.

He proceeded to make the necessary use of the file and chisel preparitory to filling, but met with great opposition from the patient, who insisted, with much feeling, that he was not operating upon the same principle the former dentist had. By a firm course, however, and appeals to her judgment, she finally consented to have him proceed on what he would term in contradistinction, "the high-pressure principle." He recommended to treat patients with kindness and forbearance, but whenever, from whim or capriece, they undertook to controle the operator and drive him from *his* position, to drive them from *theirs*, and require strict obedience to the requirements of the case in hand.

Dr. J. M. Harris was a strong friend to the file, if not direct, indirect; used cutting instruments as a substitute for the file. Had seen many teeth operated upon by many gentlemen—Drs. Hudson, Plantau, and others. Alluded to the variable character of the teeth, and thought reference should be had to this in the use of the file.

Dr. Townsend was glad the different methods of separating the teeth had been spoken of; he used cutters as preferable in many cases; used them much more than files. Thought the dentist should be an artist as well as a dentist; that he should be able to file, as well as supply teeth, with a correct eye and judgment. Was glad to see dentistry cut up in separate branches, as well calculated to improve each branch the more rapidly.

Dr. Miller used the file but very little; cut away nine-tenths with chisels and excavators; file very little between back teeth, but make generous spaces with the chisel.

Dr. Clark.—An interesting question to him; had had much difficulty in bringing patients to what was considered ultraism in the use of the file in separating. It was the "head and front" of his offending in practice, but with young persons could not assent to the use of the file for the entire separations, when the disease could be removed and the tooth preserved in near its normal shape, and a filling be inserted, retaining a perfect margin of enamel around the filling. He used wedges of India rubber, wood, or cotton, according to the case. He always used great care with children's teeth, not to produce too much inflammation. A careless use of India rubber he thought would do it.

On interrogation, said he was not able to point out the idiosyncrasies presented, under which the application of separating wedges were inadmissable, having never seen in his practice those much-talked-of cases of injury (resulting in the death of the tooth) from their use. He would say, however, that when there was a congested state of the gums, or a tendency to that state, he always had been particularly careful in the application, supposing that injury would more easily be caused by force in separating.

Dr. Flagg was not so strong an advocate of filing teeth; saved as much of a tooth as possible; used India rubber, gradually coaxing the teeth apart. His experience with India rubber had been satisfactory.

Dr. McQuillen preferred the chisels, when applicable, to the file, as by their use the operations are facilitated, and patients do not make the same objections to them. Where the teeth approximate so close to each other as to induce a doubt about the existence of decay, had found the introduction of India rubber between the teeth for a few hours of great service, by separating them sufficiently to admit of a thorough examination; had also found this an advantageous course to be pursued in operating on a class of teeth where the file or chisel must be applied, but from which a very limited portion should be removed.

Dr. John Allen filed freely; was satisfied many teeth could be saved by the use of the file properly applied.

Prof. Parry alluded to cases where two-thirds of the teeth were filed away and were saved; was favorable to the use of chisels when applicable; the practice had come into condemnation from the abuse of it.

Dr. James Locke considered the chief glory of the dentist consisted in saving the natural teeth, rather than in substitutions. The error of many young practitioners was, in filing the front teeth too liberally; he was in favor of a discriminating use of the file.

Prof. Buckingham described an instrument he used for cutting the teeth, which was somewhat in the form of cutting forceps, or Physic's forceps, and which he found very useful in certain cases.

Dr. Ballard never used a file unless compelled; used wedges of wood and India rubber in making a wide separation. When filing was indicated, the operation should be performed thoroughly.

Dr. Neall remarked, he occasionally saw the admirable operations of Hudson, Haydon, Harrington and the earlier operators, in this direction, and thought the operation of filing—its finish, the smoothing of angles, &c., &c., greatly improved. He considered filing as perfect and as important an operation in its place, as filling; when indicated, went for radical filing for the sake of space, freedom from accumulation, &c., &c. Never filed in straight, but in *curved* lines. He was very fond of an *old* file, for the sake of the good "stuff" generally to be found in it, of which could be formed cutters, chisels, &c., of every conceivable shape. They, with him, were always the forerunners of the file, using it mainly for finishing the space or surface.

Dr. Walton was in favor of a free use of the file, and gave his experience with it.

Dr. Dwinelle had used the file more freely than at present. He seldom used it in filling front cavities—used pine wedges, India rubber, cotton and other substances. Had used boiled India rubber, which was semi-elastic; dispensed with the use of the file whenever possible, yet considered it one of the most valuable instruments in a dental case; related a case where the file had been freely used twenty-five years ago, and the teeth yet in good condition.

Prof. Arthur stated that he always preferred making a permanent separation of teeth requiring filling on their proximate surfaces, to pressing them asunder in the manner advocated by the gentlemen who had preceded him. He preferred this, as the mere act of mastication did a great deal toward keeping these surfaces clean after the operation, contributing consequently a great deal toward their preservation. Upon this subject, a most important one, there was more to be said than could be said by any one on such an occasion.

Dr. Dwinelle explained, by draft upon the black board, how he would operate when the incisors were decayed on the approximal surfaces, in which case he would operate with wedges.

Dr. H. H. Martin felt much interested in the subject, and gave his experience; he had separated with the file, but had occasionally to replace the fillings when separated, with India rubber; still used it, however—but chifley the file.

Dr. Rich had tried all the known substances, except blocks of wood; was most pleased with boiled India rubber as preferable to that in the ordinary form; still used it, as the best article he knew of. In separating, used cutters freely, which had the advantage of removing the tooth-bone more rapidly and pleasantly than the file.

To an inquiry, replied that he kept the India rubber in place, by the use of metallic caps, silk, etc.

At the close of Dr. Rich's remarks, a proposition was made to change the place of next meeting from New York to Philadelphia, and various reasons in favor of the change, such as "Philadelphia was most central," "it was the child of Philadelphia," "want of unanimity in New York," and "less professional intercourse there than here," etc., etc., all of which were replied to, and other places proposed, and, after considerable discussion, the proposition was finally withdrawn by the mover.

On motion of Dr. Flagg,

*Resolved*, That this body recommend to the profession the formation of local associations.

A vote of thanks to the President, for the impartial and satisfactory manner in which he had presided over the convention, was passed unanimously, which was responded to by the President in some complimentary remarks toward those with whom it had been his pleasure and profit to associate with in his studies, his earlier practice, and on the present occasion.

On motion of Dr. Goddard, the thanks of the members residing out of the city (Philadelphia) was returned to the city members, for the kindness extended to them.

On motion, adjourned.—*News Letter.*

